# Supplementary material for: A Yap-dependent mechanoregulatory program sustains cell migration for embryo axis assembly
Source: Nat Commun. 2023 May 16;14:2804. doi: 10.1038/s41467-023-38482-w (PMC10188487; doi:10.1038/s41467-023-38482-w)
Supplement: Supplementary file 1 — Supplementary Information [file 41467_2023_38482_MOESM1_ESM.pdf]

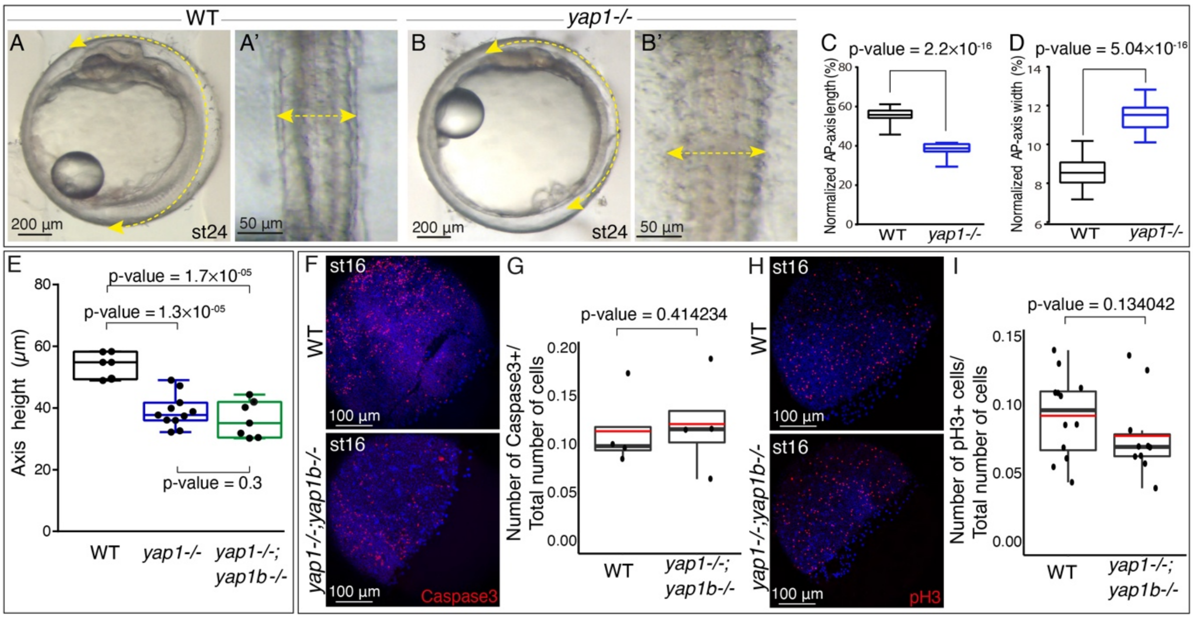

Supplementary Figure 1. Analysis of *yap* mutants' phenotype

(A, B) Brightfield images of WT and *yap1*$^{-/-}$ embryos at stage 24. Yellow double-headed arrows highlight the shortening and the widening of the A-P axis (A-B). A', B' correspond to magnifications of the axis. **(C, D)** Quantification of the normalized length (C) and width (D) of WT and *yap1*$^{-/-}$embryo A-P axis at stage 24. P-value = 2.2×10^{-16} (C), P-value = 5.04×10^{-16} (D). n= 62 WT embryos, n= 20 *yap1*$^{-/-}$embryos. Boxes represent the quartiles; the whiskers indicate the maximum and minimum values; the lines indicate the mean; WT in black and *yap1*$^{-/-}$ in blue. **(E)** Quantification of the D-V height of the axis (see Figure 1) in WT, *yap1*$^{-/-}$ and *yap1*$^{-/-}$*;yap1b*$^{-/-}$ embryos at stage 16-17. P-values are indicated in the figure. Boxes represent the quartiles; the whiskers indicate the maximum and minimum values; the lines indicate the mean; WT in black, *yap1*$^{-/-}$ in blue, *yap1*$^{-/-}$*;yap1b*$^{-/-}$ in green. Points indicate independent embryos. n= 6 WT embryos, n= 11 *yap1*$^{-/-}$ embryos, n= 7 *yap1*$^{-/-}$*;yap1b*$^{-/-}$ embryos. **(F)** Confocal images show Caspase 3+ cells in red and DAPI in blue in WT and *yap1*$^{-/-}$*;yap1b*$^{-/-}$. **(G)** Quantification of caspase 3-positive cells per total number of cells in WT and *yap1*$^{-/-}$*;yap1b*$^{-/-}$ embryos. P-value = 0.414234. n= 4 WT embryos, n=4 *yap1*$^{-/-}$*;yap1b*$^{-/-}$ embryos. **(H)** Confocal images show pH3+ cells in red and DAPI in blue in WT and *yap1*$^{-/-}$*;yap1b*$^{-/-}$. **(I)** Quantification of pH3+ cells per total number of cells in WT and *yap1*$^{-/-}$*;yap1b*$^{-/-}$ embryos. P-value = 0.134042. n= 12 WT embryos, n=10 *yap1*$^{-/-}$*;yap1b*$^{-/-}$ embryos. Boxes represent the quartiles; the whiskers indicate the maximum and minimum values. Red and black lines indicate the median and the mean, respectively. Points indicate independent embryos. Two-sided Student's t-tests were performed to evaluate statistical significance. Scales bars are 200 µm (A,B), 50 µm (A'-B') and 100 µm (F, H). Source data are provided as a Source Data file.

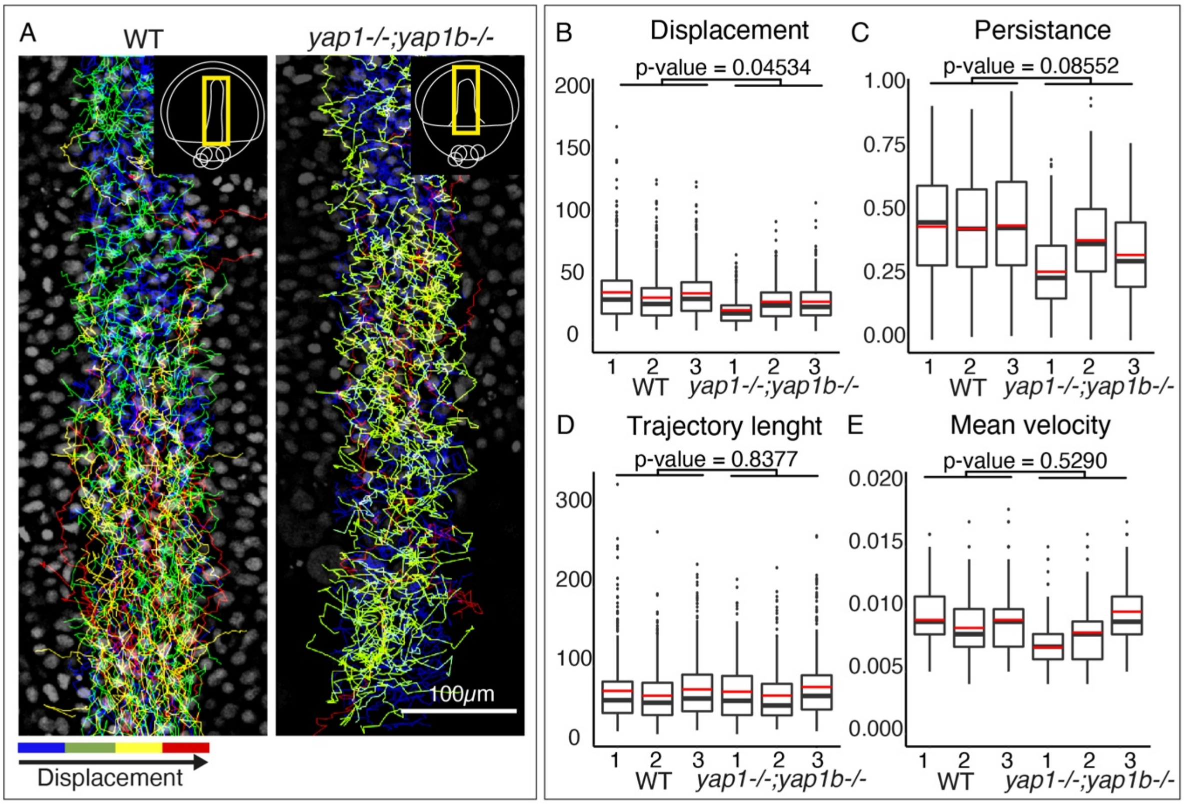

Supplementary Figure 2. Midline cell migration in gastrulating WT and *yap* mutant embryos

(A) Total individual cell migratory tracks over 8 h in the midline of WT and *yap1*$^{-/-}$;*yap1b*$^{-/-}$ embryos. The color code of the trajectory lines indicates the cells' displacement (distance between the start and end position of a cell). Displacement values were represented as: Blue = 10-20; Green = 20-40; Yellow = 40-60; Red > 60. Yellow rectangles in schematic embryo representations indicate the area depicted in each image. **(B)** Quantification of cell displacement in WT and *yap1*$^{-/-}$;*yap1b*$^{-/-}$ embryos. P-value = 0.04534. **(C)** Quantification of cell migratory persistence, measuring for how long a cell keeps the same direction of movement, in WT and *yap1*$^{-/-}$;*yap1b*$^{-/-}$ embryos. P-value 0.08552. **(D)** Quantification of cell trajectory length, measuring the total length of a cell trajectory. P-value = 0.8377. **(E)** Quantification of the cell mean velocity, measuring distance between two cells' positions divided by the time difference, in WT and *yap1*$^{-/-}$;*yap1b*$^{-/-}$ embryos. P-value = 0.5290. Boxes represent the quartiles; the whiskers indicate the maximum and minimum values. Red and black lines indicate the median and the mean, respectively. To analyze whether experimental groups were significantly different, a variance test followed by a two-sided Student's t-tests were performed on the means of WT and *yap1*$^{-/-}$;*yap1b*$^{-/-}$ embryos (n= 3 embryos; n= 416 cells were examined in WT1, n= 401 cells were examined in WT2, n= 516 cells were examined in WT3, n= 271 cells were examined in *yap1*$^{-/-}$;*yap1b*$^{-/-}$ mutant 1, n= 295 cells were examined in *yap1*$^{-/-}$;*yap1b*$^{-/-}$ mutant 2, n= 346 cells were examined in *yap1*$^{-/-}$;*yap1b*$^{-/-}$ mutant 3). Scale bars are 100 µm(A). Source data are provided as a Source Data file.

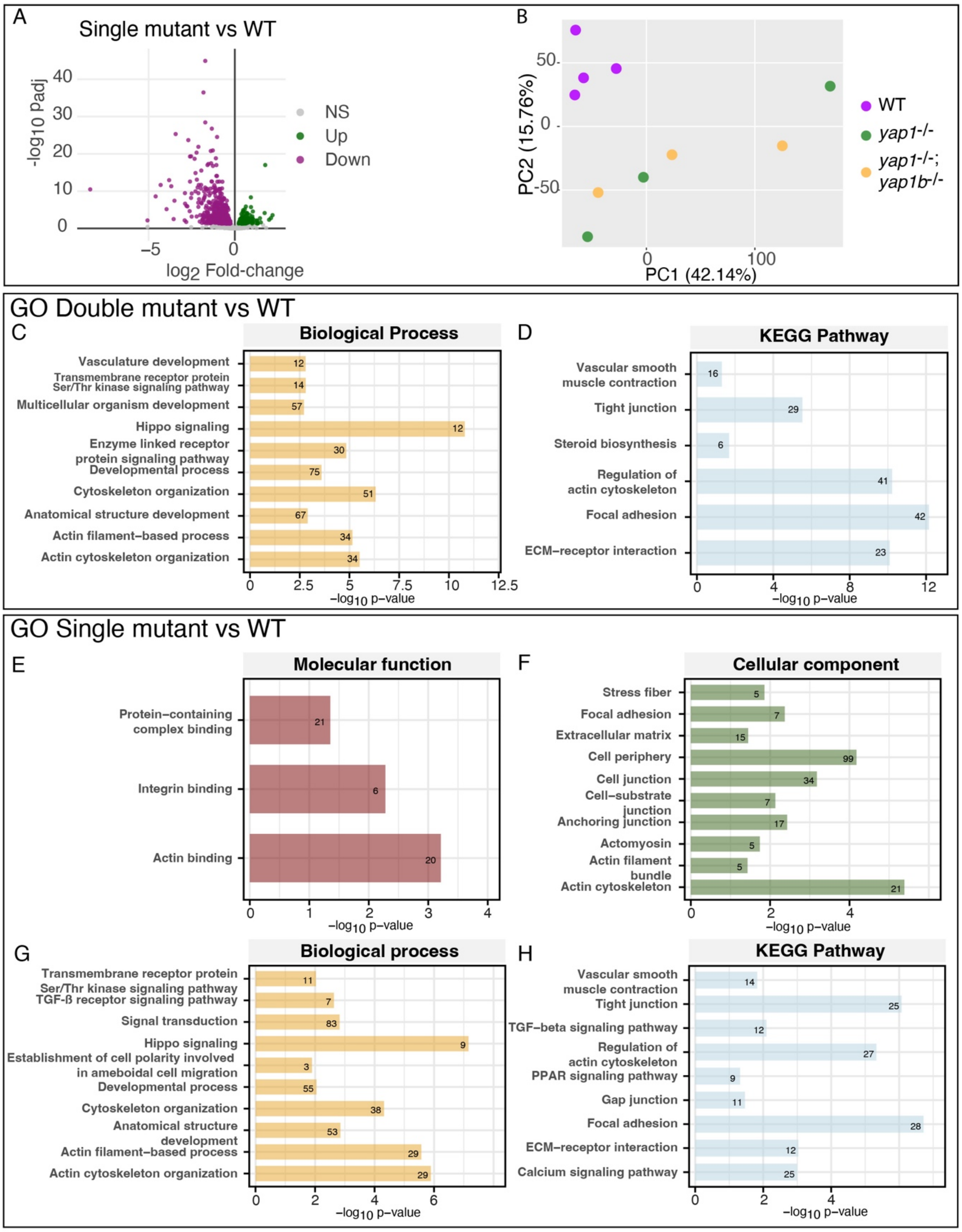

Supplementary Figure 3. Characterization of Yap-dependent transcriptional programs

(A) Volcano plot graph showing DEGs between WT and *yap1*$^{-/-}$ embryos. Gray dots: no differentially expressed genes; Green dots: up-regulated genes in *yap1*$^{-/-}$ embryos compared with WT; Magenta dots: down-regulated genes in *yap1*$^{-/-}$ embryos compared with WT. Differential gene expression analysis was carried out using the R package DESeq2, using by default Wald test and Benjamini-Hochberg correction (padj < 0.05; -log FC = 1). **(B)** PCA graph showing the RNA-seq data variability between WT, *yap1*$^{-/-}$, and *yap1*$^{-/-}$*;yap1b*$^{-/-}$ embryos. **(C-D)** Gene Ontology (GO) enrichment of the DEGs in *yap1*$^{-/-}$*;yap1b*$^{-/-}$ embryos compared with WT, classified in Biological processes (C) and KEGG Pathway (D). **(E-H)** Gene Ontology (GO) enrichment of the DEGs in *yap1*$^{-/-}$ embryos compared with WT, classified in molecular function (E), cellular component (F), biological processes (G) and KEGG Pathway (H). gProfiler was used for this analysis (see Methods section). Source data are provided as a Source Data file.

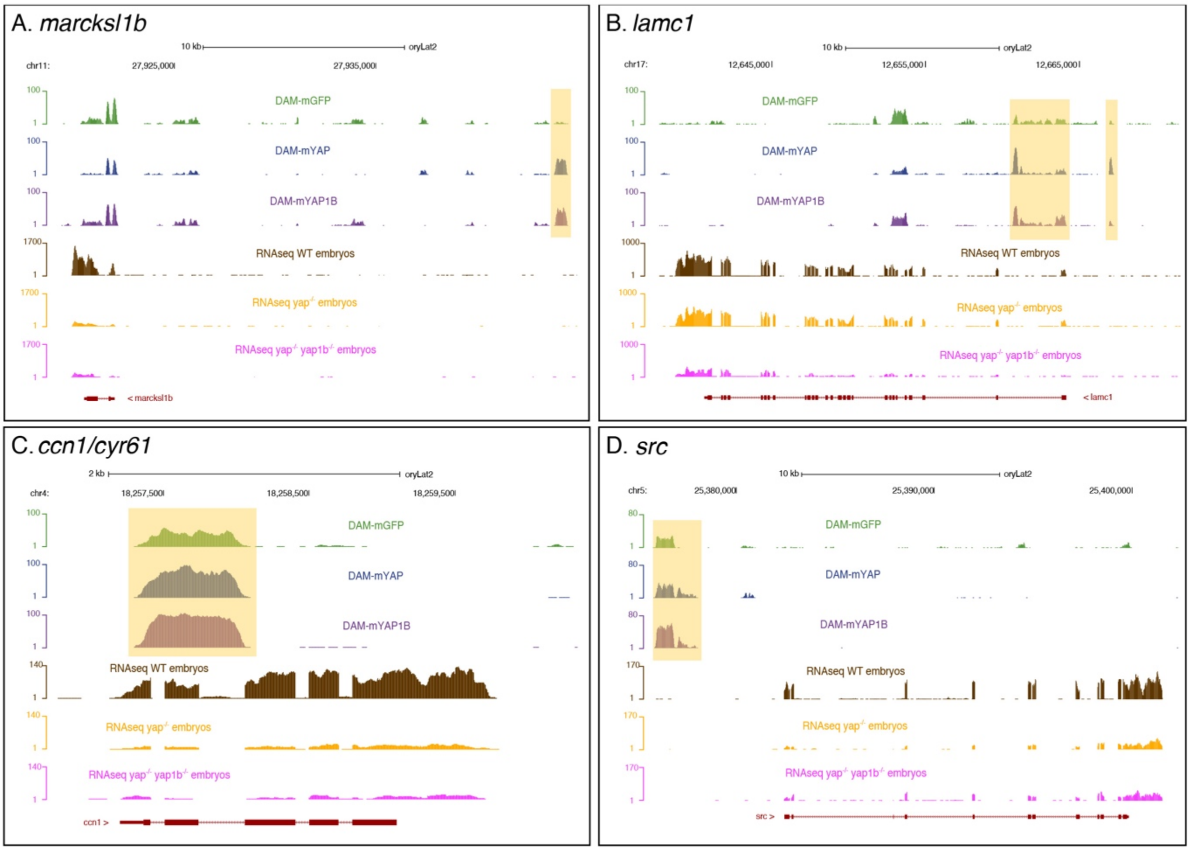

Supplementary Figure 4. Binding of Yap1/Yap1b to the chromatin is associated to significant DEGs

Overview of Yap1 and Yap1b DamID-seq tracks (Vazquez-Marin et al., 2019), as well as RNA-seq profiling of WT and *yap* single and double mutants for four representative loci: *marcksl1b* (A), *lamc1* (B), ccn2/cyr61 (C) and src (D). Specific binding of Yap1 and Yap1b to the genome is highlighted in yellow.

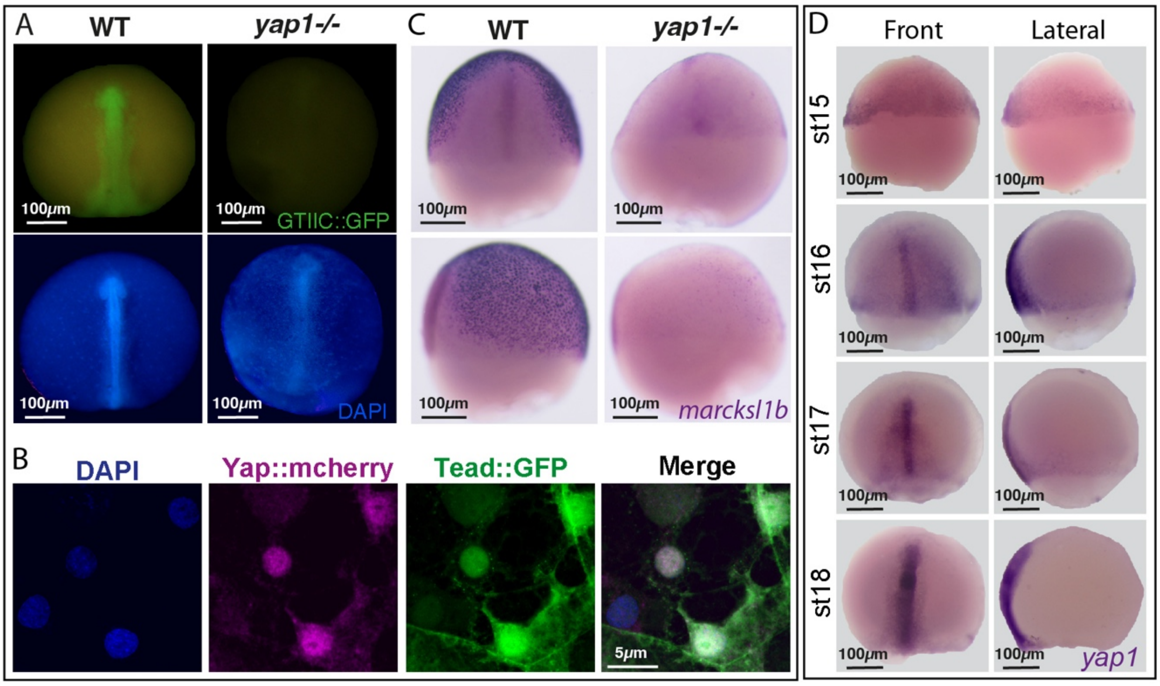

Supplementary Figure 5. Yap activity dynamics during gastrulation

(A) Activity of the Tead/Yap sensor (*GTIIC::GFP)* in WT and *yap1*$^{-/-}$ transgenic embryos shown under the fluorescent stereo microscope at stage 18. DAPI staining of these embryos is also shown. **(B)** DAPI counterstained confocal images of dorsally converging cells from *GTIIC::GFP* transgenic embryos injected with *yap1*::mCherry. Blue channel: DAPI; Magenta channel: *yap1*::mCherry signal; Green channel: GFP signal. **(C)** ISH analysis of the expression of *marcksl1b* in WT and *yap1*$^{-/-}$ embryos at stage 16. Front and lateral images are shown. **(D)** Whole mount *ISH* showing *yap1* expression at stages 15, 16, 17 and 18 in medaka embryos. Front and lateral images are shown. Scale bars 100 µm (A, C, D) and 5 µm (B).

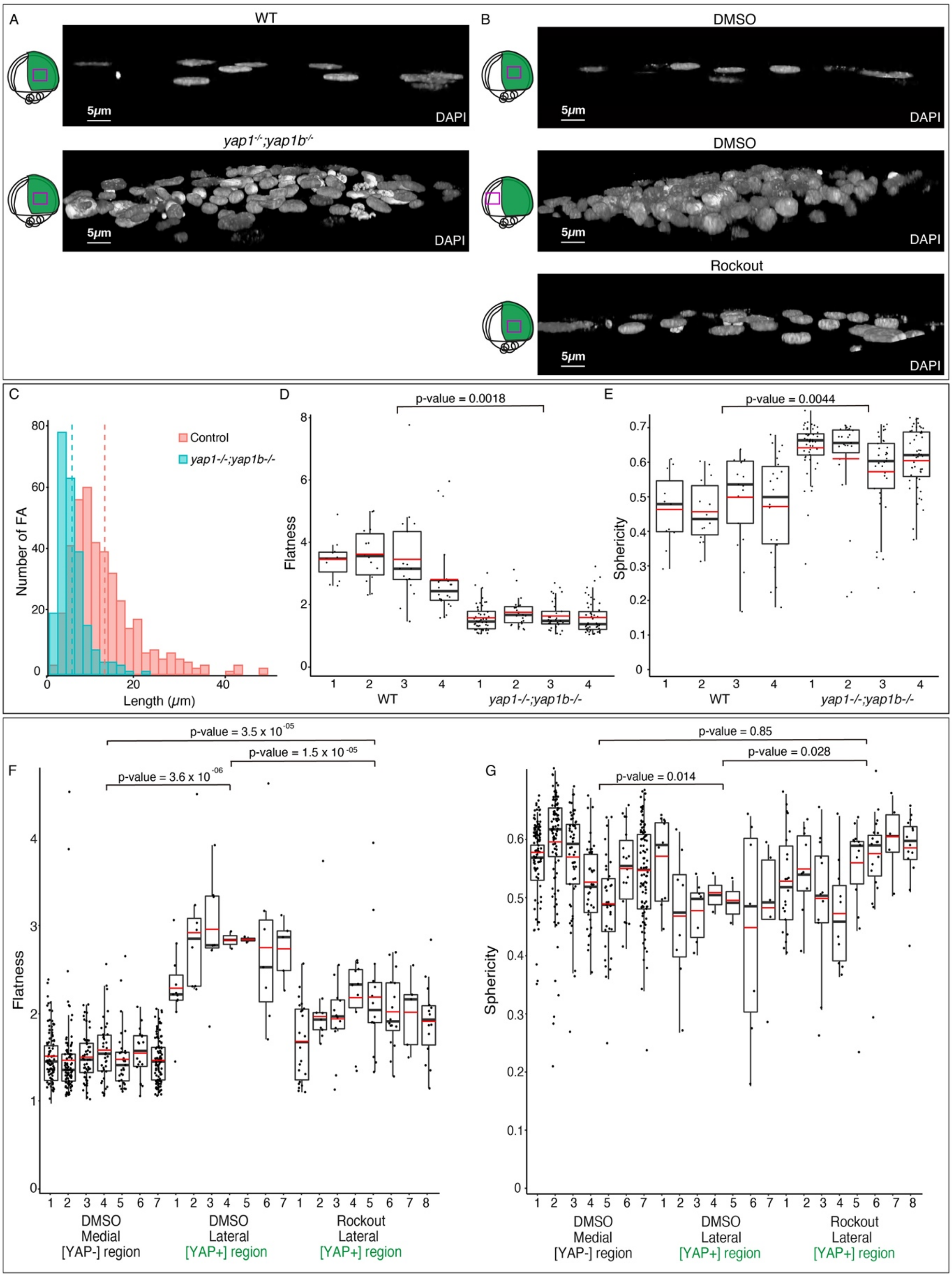

Supplementary Figure 6. Nuclear morphology in mutant and Rockout treated cells

(A) Maximum projection of DAPI stained nuclei (lateral) in stage 16 WT and *yap1-/-;yap1b-/-* embryos (see movie S4). **(B)** Maximum projection of DAPI stained nuclei in stage 16 embryos treated with DMSO (lateral and medial) or Rockout (lateral) (see movie S5). **(C)** Distribution of focal adhesions according to their length in WT and *yap1-/-;yap1b-/-* embryos. Red line: mean of focal adhesions length in WT embryos. Blue line: mean of focal adhesions length in *yap1-/-;yap1b-/-* embryos. **(D-E)** Quantification of nuclei flatness (D), average values are shown in figure 6F, and sphericity (D) for individual stage 16 WT and *yap1-/-;yap1b-/-* embryos. n= 4 WT embryos, n= 4 *yap1-/-;yap1b-/-* embryos. **(F-G)** Quantification of nuclei flatness (F), average values are shown in figure 7D, and sphericity (G) for individual stage 16

embryos treated with DMSO (lateral and medial) or Rockout (lateral). P-value are indicated in the figure. n= 7 DMSO treated embryos examined in medial regions, n= 7 DMSO treated embryos examined in lateral regions, n= 8 Rockout treated embryos. Boxes represent the quartiles; the whiskers indicate the maximum and minimum values. Red and black lines indicate the median and the mean, respectively. Points indicate cells. Two-sided Student's t-tests were performed to evaluate statistical significance. Scale bars are 5 µm (A-B). Source data are provided as a Source Data file.

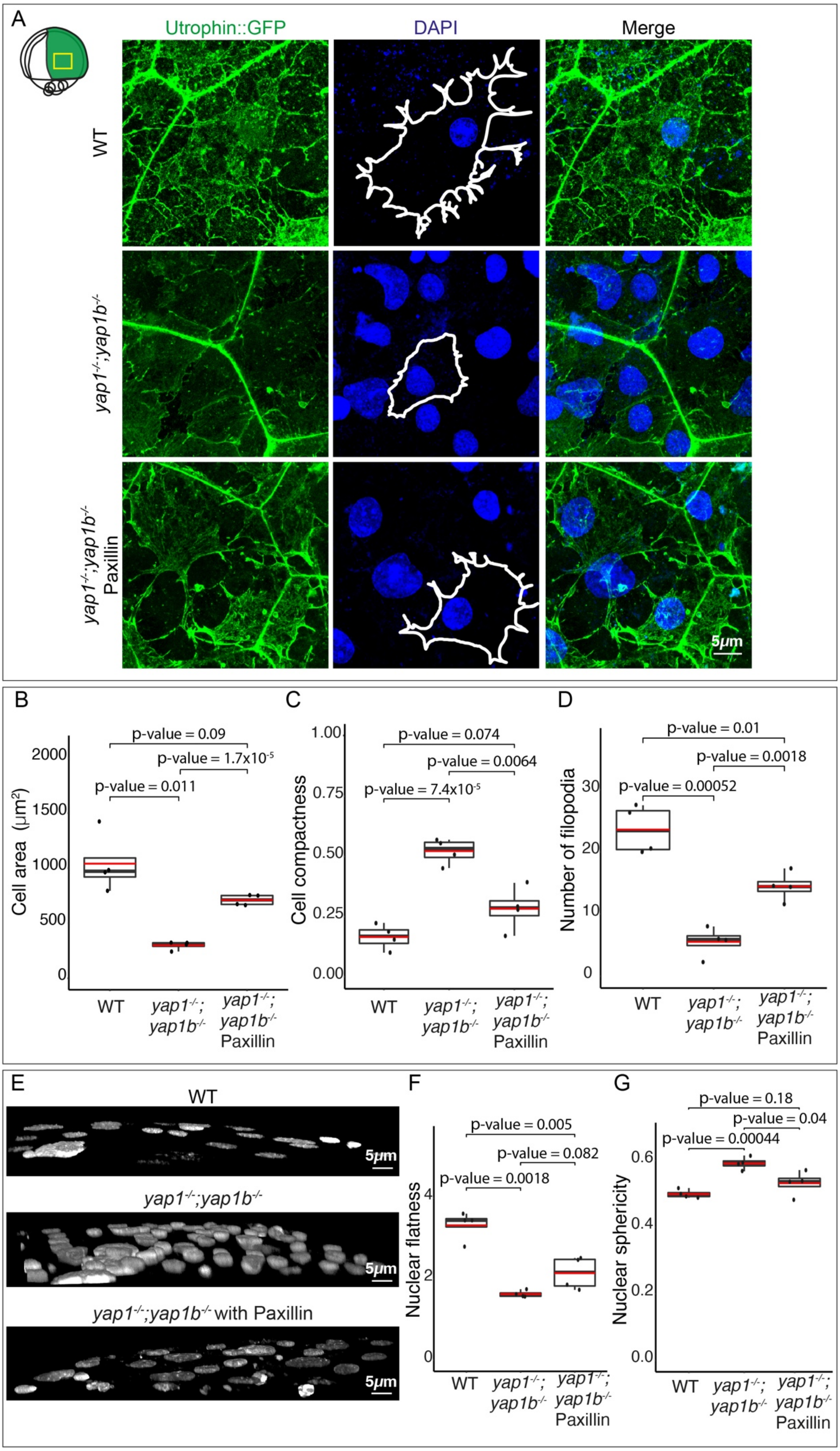

A
Utrophin::GFP
DAPI
Merge
WT
yap1-/-;yap1b-/-
yap1-/-;yap1b-/- Paxillin
5μm
B
C
D
Cell area (μm²)
p-value = 0.09
p-value = 1.7x10-5
p-value = 0.011
Cell compactness
p-value = 0.074
p-value = 0.0064
p-value = 7.4x10-5
Number of filopodia
p-value = 0.01
p-value = 0.0018
p-value = 0.00052
WT
yap1-/-; yap1b-/-
yap1-/-; yap1b-/- Paxillin
E
F
G
WT
yap1-/-;yap1b-/-
yap1-/-;yap1b-/- with Paxillin
Nuclear flatness
p-value = 0.005
p-value = 0.082
p-value = 0.0018
Nuclear sphericity
p-value = 0.18
p-value = 0.04
p-value = 0.00044

Supplementary Figure 7. Paxillin overexpression rescues cell and nuclear morphology in *yap* mutant embryos

(A) Confocal microscopy images of dorsally converging cells from WT, *yap1*-/-;*yap1b*-/- and Paxillin-rescued *yap1*-/-;*yap1b*-/- embryos injected with *Utrophin::GFP* and stained with DAPI. Schematic representation of the embryo indicating the area of interest with a yellow rectangle is shown in the upper left side. Cell shapes are represented with white lines in the images corresponding to DAPI. **(B)** Quantification of average cell area in WT, as well as in *yap1*-/-;*yap1b*-/- and Paxillin-rescued *yap1*-/-;*yap1b*-/- embryos. **(C)** Quantification of average cell compactness, as determined by the ratio between the cell area and the area of the circle having the same perimeter, in WT, *yap1*-/-;*yap1b*-/- and Paxillin-rescued *yap1*-/-;*yap1b*-/- embryos. **(D)** Quantification of the average number of filopodia in WT, *yap1*-/-;*yap1b*-/- and Paxillin-rescued *yap1*-/-;*yap1b*-/- embryos. **(E)** Maximum projection of DAPI stained nuclei (lateral) in stage 16 WT, *yap1*-/-;*yap1b*-/- and Paxillin-rescued *yap1*-/-;*yap1b*-/- embryos. **(F)** Quantification of average nuclei flatness, which refers to the ratio between the second and the third axis of an ellipsoid, in WT, *yap1*-/-;*yap1b*-/- and Paxillin-rescued *yap1*-/-;*yap1b*-/- embryos. **(G)** Quantification of average nuclei sphericity in WT, *yap1*-/-;*yap1b*-/- and Paxillin-rescued *yap1*-/-;*yap1b*-/- embryos. Boxes represent the quartiles; the whiskers indicate the maximum and minimum values. Red and black lines indicate the median and the mean, respectively. Points indicate independent embryos. n= 4 embryos. P-values are indicated in the figure. Two-sided Student's t-tests were performed to evaluate statistical significance. Scale bars = 5 µm. Source data are provided as a Source Data file.

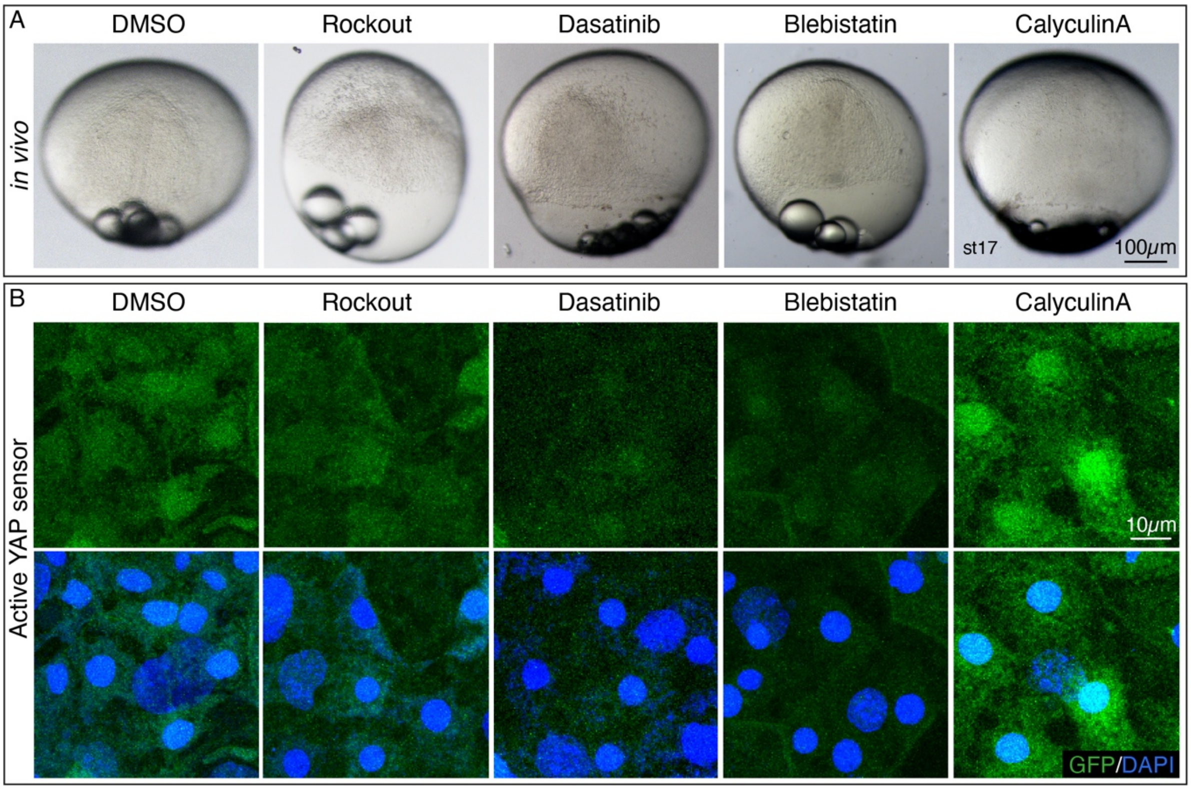

Supplementary Figure 8. Gastrulation phenotype and Yap activity under intracellular tension alterations

(A) Brightfield images of stage 17 WT embryos treated with DMSO, Rockout, Dasatinib, Blebistatin or CalyculinA during 4 h. Frontal stereo microscope images of embryos are shown. **(B)** Confocal microscopy images of dorsally converging cells stained with DAPI from stage 16 transgenic embryos for the Tead/Yap sensor *GTIIC::GFP* treated with DMSO, Rockout, Dasatinib, Blebistatin or CalyculinA for 2 h. Scale bars are 100 µm (A) and 10 µm (B).

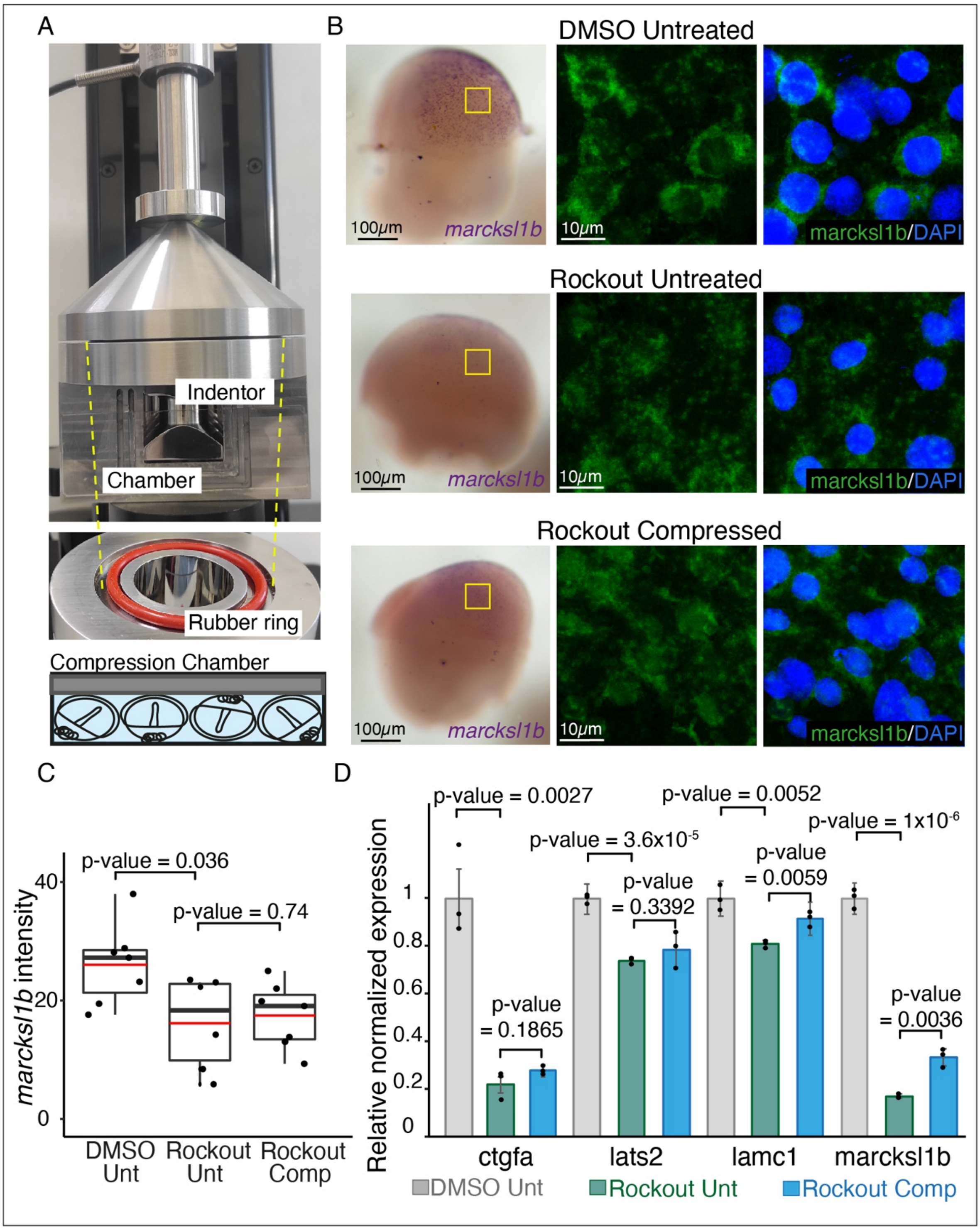

Supplementary Figure 9. Rho/ROCK-dependent response of Yap activity to compression

(A) Motorized mechanical tester (Univert), equipped with a customized chamber for embryo compression. **(B)** ISH analysis distribution of *marcksl1b* in DMSO untreated (not compressed), Rockout untreated and Rockout compressed WT embryos during 20 min. Lateral stereo microscope images are shown. Confocal microscopy images of *marcksl1b* fluorescent ISH stained with DAPI from the sections indicated with yellow rectangles are shown. **(C)** Quantification of *marcksl1b* fluorescent ISH signal intensity in DMSO untreated, Rockout untreated and Rockout compressed embryos. P-values are indicated in the figure. Boxes represent the quartiles; the whiskers indicate the maximum and minimum values. Red and black lines indicate the median and the mean, respectively. Points indicate independent embryos. n= 7 DMSO untreated embryos, n= 6 Rockout untreated embryos, n= 7 Rockout compressed embryos. **(D)** mRNA levels of *ctgfa*, *lats2*, *lamc1* and *marcksl1b* in DMSO untreated, Rockout untreated and Rockout compressed embryos as quantified by RT-qPCR. P-values are indicated in the figure. Data are represented as mean ± SD; points indicate technical

replicates. n= 20 embryos. Two-sided Student's t-tests were performed to evaluate statistical significance. Scale bars are 100 µm and 10 µm (B). Source data are provided as a Source Data file.

Gene ID	Gene name	log2FoldChange	GeneOntology
ENSORLG00000027007	mfap4	-1,99	Extracellular Matrix
ENSORLG00000011273	col1a1b	-1,87	Extracellular Matrix
ENSORLG00000010226	angpt1	-1,85	Extracellular Matrix
ENSORLG00000010756	ccn1	-1,62	Integrin Binding, Extracellular Matrix
ENSORLG00000008004	actr2b	-1,62	Cytoskeleton Organization
ENSORLG00000018064	ccn2a	-1,59	Integrin Binding, Extracellular Matrix
ENSORLG00000014159	marcksl1b	-1,53	Cytoskeleton Organization
ENSORLG00000003389	itgb3a	-1,35	Integrin Binding, Focal Adhesion
ENSORLG00000008993	ptk2bb	-1,23	Focal Adhesion
ENSORLG00000024658	arhgef19	-1,14	Cytoskeleton Organization
ENSORLG00000020765	itgb4	-1,12	Integrin Binding, Focal Adhesion
ENSORLG00000004319	col1a2	-1,04	Extracellular Matrix
ENSORLG00000009834	fermt1	-1,01	Integrin Binding, Focal Adhesion
ENSORLG00000013126	unknown	-1,01	Cytoskeleton Organization
ENSORLG00000016273	amotl2b	-0,96	Hippo Signalling, Cytoskeleton Organization
ENSORLG00000024032	marcksl1a	-0,95	Cytoskeleton Organization
ENSORLG00000009847	smtnl	-0,94	Cytoskeleton Organization
ENSORLG00000002553	tead4	-0,93	Hippo Signalling
ENSORLG00000009876	muc17	-0,92	Extracellular Matrix
ENSORLG00000004966	ADAMTS5	-0,85	Extracellular Matrix
ENSORLG00000007439	tead1b	-0,84	Hippo Signalling
ENSORLG00000029189	tuba1l2	-0,84	Cytoskeleton Organization
ENSORLG00000002708	yap1	-0,83	Hippo Signalling
ENSORLG00000016655	lima1a	-0,82	Cytoskeleton Organization
ENSORLG00000014658	col5a2a	-0,82	Extracellular Matrix
ENSORLG00000012987	tubb5	-0,81	Cytoskeleton Organization
ENSORLG00000015488	bmper	-0,80	Extracellular Matrix
ENSORLG00000002046	coro1a	-0,79	Cytoskeleton Organization
ENSORLG00000001953	NCKAP5	-0,78	Cytoskeleton Organization
ENSORLG00000001946	unknown	-0,78	Extracellular Matrix
ENSORLG00000019677	lama5	-0,77	Integrin Binding, Extracellular Matrix
ENSORLG00000000178	adamtsl5	-0,77	Extracellular Matrix
ENSORLG00000015895	sptb	-0,75	Cytoskeleton Organization
ENSORLG00000000761	vwa1	-0,73	Extracellular Matrix
ENSORLG00000012731	rock2b	-0,72	Cytoskeleton Organization
ENSORLG00000024836	map1ab	-0,72	Cytoskeleton Organization
ENSORLG00000005263	svild	-0,69	Cytoskeleton Organization
ENSORLG00000017032	tpm4a	-0,69	Cytoskeleton Organization
ENSORLG00000005664	col11a1a	-0,68	Extracellular Matrix
ENSORLG00000018013	ccn6	-0,64	Integrin Binding, Extracellular Matrix
ENSORLG00000000815	evpla	-0,64	Cytoskeleton Organization
ENSORLG00000015065	evplb	-0,64	Cytoskeleton Organization
ENSORLG00000003176	coro6	-0,63	Cytoskeleton Organization
ENSORLG00000009740	myo1cb	-0,63	Cytoskeleton Organization
ENSORLG00000026496	synpo	-0,63	Cytoskeleton Organization
ENSORLG00000012150	MYO1D	-0,62	Cytoskeleton Organization
ENSORLG00000015457	amotl2a	-0,62	Hippo Signalling, Cytoskeleton Organization
ENSORLG00000018617	fgl1	-0,61	Extracellular Matrix
ENSORLG00000005573	yap1b	-0,61	Hippo Signalling
ENSORLG00000029757	wtip	-0,60	Hippo Signalling, Cytoskeleton Organization
ENSORLG00000025663	paplna	-0,59	Extracellular Matrix
ENSORLG00000004990	adamts1	-0,56	Extracellular Matrix
ENSORLG00000019746	cav1	-0,54	Focal Adhesion
ENSORLG00000023311	plecb	-0,53	Focal Adhesion, Citoskeleton Organization
ENSORLG00000018677	pard6a	-0,53	Cytoskeleton Organization
ENSORLG00000008336	PKP1	-0,53	Cytoskeleton Organization
ENSORLG00000007419	lamc1	-0,52	Extracellular Matrix
ENSORLG00000014111	pdlim1	-0,52	Cytoskeleton Organization
ENSORLG00000019702	pacsin3	-0,51	Cytoskeleton Organization
ENSORLG00000001861	pard6b	-0,50	Cytoskeleton Organization

Supplementary Table 1: List of significant DEGs between WT and yap1-/-;yap1b-/- stage 16 embryos (FC ≤ -0.5) linked to most significant GO terms identified in the comparative RNA-seq analysis (see Figure 3).

Gene name	Primers (5'-3')
no-tail (ntl)	AGAGTTAACCAACGAGATGATCG
	CAACCTCCAAGTTGGGAGTATC
Goosecoid (gsc)	GTTCAGCATCGACAGCATCTTG
	TTTTCCTCCCTCAAATGGACCTT
sox3	TGATGGAAACCGAGATCAAGACC
	GAGGACATCATAGGGTACTGCAG
yap1	ACTCCAGATGACTTCCTCAACAG
	AGCCTTGAAGACACAGACACAAT
marcksl1b	ATGGGATCCCAGTCATCCAAGGG
	ACTCCACCCTGCTTCACCTCATGG
***ef1a* (qPCR)**	AAACCCAGAAACACCGAAACAT
	CCTCCGCACTTGTAGATCAG
***ctgfa* (qPCR)**	GCCGACAGGAGATCCACTTG
	CCTGCAGCCGCGTATGAGTA
***cyr61* (qPCR)**	GAGCTCTCCCTGCCCAATTT
	CTGTATGCAGGCAGGGTCTT
***lats2* (qPCR)**	AACGAGCAACTTTTTCCCGC
	CATCTGCGCCATGTGTGATG
***lmc1* (qPCR)**	GAGCGCAGAGTGTTACTTCG
	GAGGAGACAGAGAGCCGATG
***marcksl1b* (qPCR)**	AAGACCAACGGACAGGAGAAC
	CTTCGGGTTTGGTGGCTTCC

Supplementary Table 2: List of primers used to generate the RNA probes.

Figure	Independent experiments
Figure 1A-C	20
Figure 1D-F	3
Figure 2A-C	3
Figure 3E	4
Figure 4B-C	6
Figure 5B	4
Figure 6A	4
Figure 6B	5
Figure S5A	6
Figure S5B	3
Figure S5C	4
Figure S5D	3
Figure 8A	4
Figure 8B	3

Supplementary Table 3: Number of experiments repeated independently.
