# Supplementary material for: A Yap-dependent mechanoregulatory program sustains cell migration for embryo axis assembly
Source: Nat Commun. 2023 May 16;14:2804. doi: 10.1038/s41467-023-38482-w (PMC10188487; doi:10.1038/s41467-023-38482-w)
Supplement: Supplementary file 3 — Description of Additional Supplementary Files [file 41467_2023_38482_MOESM3_ESM.pdf]

Description of Additional Supplementary Files

File Name: Supplementary Data 1
Description: Differentially expressed genes (DEGs) in yap single and double mutants compared to WT embryos.

File Name: Supplementary Data 2
Description: Enriched Gene Ontology (GO) terms associated to DEGs in yap double mutants vs WT.

File Name: Supplementary Data 3
Description: Differentially expressed genes (DEGs) in yap double mutants (vs WT) associated to enriched GO terms.

File Name: Supplementary Movie 1
Description: Tridimensional (3D) reconstruction of DAPI (blue) and Phalloidin (green) immunostained posterior axis WT, yap1-/-, and yap1-/-;yap1b-/- stage 17 embryos.

File Name: Supplementary Movie 2
Description: Cell tracking trajectories (8 hours) of dorsally converging precursors in WT, yap1-/-, and yap1-/- ;yap1b-/- embryos injected with Histone2b::GFP for nuclei visualization.

File Name: Supplementary Movie 3
Description: Time-lapse confocal imaging of WT GTIIC::GFP transgenic embryos.

File Name: Supplementary Movie 4
Description: Tridimensional (3D) reconstruction of DAPI stained nuclei in stage 16 WT and yap1-/-;yap1b-/- embryos.

File Name: Supplementary Movie 5
Description: Tridimensional (3D) reconstruction of DAPI stained nuclei (stage 16) at medial and lateral regions of a DMSO treated embryo, as well as at lateral region of Rockout treated embryo.
