# Supplementary material for: A Yap-dependent mechanoregulatory program sustains cell migration for embryo axis assembly
Source: Nat Commun. 2023 May 16;14:2804. doi: 10.1038/s41467-023-38482-w (PMC10188487; doi:10.1038/s41467-023-38482-w)
Supplement: Supplementary file 12 — Reporting Summary [file 41467_2023_38482_MOESM12_ESM.pdf]

nature portfolio

Corresponding author(s): Juan R. Martinez-Morales
María Almuedo- Castillo

Last updated by author(s): April, 24, 2023

Reporting Summary

Nature Portfolio wishes to improve the reproducibility of the work that we publish. This form provides structure for consistency and transparency in reporting. For further information on Nature Portfolio policies, see our Editorial Policies and the Editorial Policy Checklist.

Statistics

For all statistical analyses, confirm that the following items are present in the figure legend, table legend, main text, or Methods section.

n/a	Confirmed	
☐	☒	The exact sample size (*n*) for each experimental group/condition, given as a discrete number and unit of measurement
☐	☒	A statement on whether measurements were taken from distinct samples or whether the same sample was measured repeatedly
☐	☒	The statistical test(s) used AND whether they are one- or two-sided<br>*Only common tests should be described solely by name; describe more complex techniques in the Methods section.*
☒	☐	A description of all covariates tested
☒	☐	A description of any assumptions or corrections, such as tests of normality and adjustment for multiple comparisons
☐	☒	A full description of the statistical parameters including central tendency (e.g. means) or other basic estimates (e.g. regression coefficient) AND variation (e.g. standard deviation) or associated estimates of uncertainty (e.g. confidence intervals)
☐	☒	For null hypothesis testing, the test statistic (e.g. *F*, *t*, *r*) with confidence intervals, effect sizes, degrees of freedom and *P* value noted<br>*Give P values as exact values whenever suitable.*
☒	☐	For Bayesian analysis, information on the choice of priors and Markov chain Monte Carlo settings
☒	☐	For hierarchical and complex designs, identification of the appropriate level for tests and full reporting of outcomes
☒	☐	Estimates of effect sizes (e.g. Cohen's *d*, Pearson's *r*), indicating how they were calculated

Our web collection on statistics for biologists contains articles on many of the points above.

Software and code

Policy information about availability of computer code

Data collection: Confocal microscopy data were collected with ZENBlack v2-1.sp3 (for Zeis 880) and LAS AF v2.7.3.9723 (Leica SP5)

Data analysis: For data analysis we used the following packages and online tools (all of them described in published literature as indicated in the methods section):
- Imaging tools: ImageJ/Fiji (version 3.96.3/v65); Plugins: TrackMate 6.0.1, Volume viewer 2.01.2, Segmentation SCF FIJI Plugins 1.2.0, 3D plugin: mcib3d_plugins-3.96.3, 3D_Viewer-4.0.3); R (version 11.453). In cell tracking experiments semi-automatic cell tracking was performed using the Fiji plugin TrackMate (blob diameter = 7.9 and threshold = 0.23). For quantification of cell morphological parameters we used the FIJI plugin MorphoLibJ 1.4.2.1. Imaris 8.02. was used for 3D rendering.
- Tools for RNAseq analysis: Trimmomatic v0.39; sortmerna v2.1; Hisat2 v2.1; samtools v0.1.19-96b5f2294a; DEBrowser v1.14.2 (uncluding ComBat and DESeq2); GProfiler v.e108_eg55_p17_39cdea3; Bedtools v2.21.0.
- Statistical tests: R (version 11.453).

For manuscripts utilizing custom algorithms or software that are central to the research but not yet described in published literature, software must be made available to editors and reviewers. We strongly encourage code deposition in a community repository (e.g. GitHub). See the Nature Portfolio guidelines for submitting code & software for further information.

Data

Policy information about availability of data

All manuscripts must include a data availability statement. This statement should provide the following information, where applicable:

- Accession codes, unique identifiers, or web links for publicly available datasets
- A description of any restrictions on data availability
- For clinical datasets or third party data, please ensure that the statement adheres to our policy

Source data are provided with this paper. RNA-seq datasets are available in the Gene Expression Omnibus (GEO) repository (https://www.ncbi.nlm.nih.gov/geo) under the following accession number: GSE201791. As a reference medaka genome (Japanese medaka HdrR; Oryzias latipes) we are using the data deposited in ENSEMBL (https://www.ensembl.org/index.html) under the accession number: ASM223467v1.

Human research participants

Policy information about studies involving human research participants and Sex and Gender in Research.

Reporting on sex and gender	n.a.
Population characteristics	n.a.
Recruitment	n.a.
Ethics oversight	n.a.

Note that full information on the approval of the study protocol must also be provided in the manuscript.

Field-specific reporting

Please select the one below that is the best fit for your research. If you are not sure, read the appropriate sections before making your selection.

☒ Life sciences ☐ Behavioural & social sciences ☐ Ecological, evolutionary & environmental sciences

For a reference copy of the document with all sections, see nature.com/documents/nr-reporting-summary-flat.pdf

Life sciences study design

All studies must disclose on these points even when the disclosure is negative.

Sample size	No sample size calculations were performed. For RNA-seq experiments we included 3 replicates, which is a minimum standard chosen to support meaningful conclusions (Soneson & Delorenzi 2013 BMC Bioinformatics), while adapting to resource and animal use constraints. The correlation coefficients among samples in these experiments were higher than 0.97. For imaging and functional experiments, sample sizes provided in the figure legends (e.g. qPCR experiments n=3) were experimentally chosen to adapt to the variance of each measured parameter.
Data exclusions	No data were excluded from the analyses.
Replication	All experimental findings were reliably reproduced. The number of replicates was 3 for RNA-seq experiments. These are the standard number of replicates for NGS experiments. In all cases correlation coefficients among samples were higher than 0.97. All replication attempts were successful. Three independent replicates were used for qPCR experiments (all replications attempts were successful). For cell tracking experiments, cells (n) were measured in a minimum of 3 independent embryos (N) for each condition. For cell tracking, cell and embryo shape measurements, N and n values are indicated in the corresponding figure legends.
Randomization	For experiments included in this article randomization occurs naturally with embryo sampling. For RNA-seq experiments, genotyped samples derive from at least 10 synchronized embryos produced by multiple random crosses.
Blinding	Given the descriptive outcome of OMICS experiments, no blinding strategy was applied: i.e. both data collection and analysis were automatic and therefore could not be influenced by any researcher bias.<br>For imaging experiments, the experimenter was blind to the genotype of the embryos. Cell tracking, embryo measurements and stainings, and cell shape parameters were performed for each embryo before genotyping.<br>For drug treatment, yolk extraction, and embryo compression experiments, the experimenter was also blind to the treatment of the embryos before measuring the different parameters.

Reporting for specific materials, systems and methods

We require information from authors about some types of materials, experimental systems and methods used in many studies. Here, indicate whether each material, system or method listed is relevant to your study. If you are not sure if a list item applies to your research, read the appropriate section before selecting a response.

Materials & experimental systems

n/a	Involved in the study
☐	☒ Antibodies
☒	☐ Eukaryotic cell lines
☒	☐ Palaeontology and archaeology
☐	☒ Animals and other organisms
☒	☐ Clinical data
☒	☐ Dual use research of concern

Methods

n/a	Involved in the study
☒	☐ ChIP-seq
☒	☐ Flow cytometry
☒	☐ MRI-based neuroimaging

Antibodies

Antibodies used	Primary antibodies:<br>anti-active caspase-3 antibody (BD Biosciences, 559565)<br>anti-phospho-Histone H3 (Ser10) antibody (Millipore 06-570)<br><br>Secondary and conjugate antibodies:<br>Alexa Fluor TM 555 Goat anti-rabbit antibody (Invitrogen #A32727)<br>anti-Digoxigenin-POD (11207733910, Roche)<br>anti-Digoxigenin-AP (11093274910, Roche)
Validation	Antibody stainings were validated both by omitting the primary antibody and by asessing their staining pattern. Primary antibodies used are well established and their staining patterns reported in the literature.

Animals and other research organisms

Policy information about studies involving animals; ARRIVE guidelines recommended for reporting animal research, and Sex and Gender in Research

Laboratory animals	The following medaka (Oryzias latipes) strains were used.<br>- iCab (wild-type) strains, the<br>- transgenic lines tg(4xGTIIc:eGFP)<br>- mutant strains yap1Δ7pb and yap1bΔ136pb<br>In all cases 4 to 8 months reproductive animals were used for crossing.
Wild animals	The study did not involve wild animals
Reporting on sex	This work concerns very early stages (15 to 24) of development in medaka embryos. Gonadal differentiation starts at stage 33 (4dpf) in this species. Therefore sex-based analyses were not performed, neither are they relevant for this work.
Field-collected samples	The study did not involved samples collected from the field.
Ethics oversight	Animal experiments were carried out according to ethical regulations. Experimental protocols have been approved by the Animal Experimentation Ethics Committees at the Pablo de Olavide University and CSIC (license number 02/04/2018/041).

Note that full information on the approval of the study protocol must also be provided in the manuscript.
